# Browsing for food: Will COVID‐induced online grocery delivery persist?

**DOI:** 10.1111/rsp3.12542

**Published:** 2022-06-01

**Authors:** Hannah Younes, Robert B. Noland, Wenwen Zhang

**Affiliations:** ^1^ Alan M. Voorhees Transportation Center, Bloustein School of Planning and Public Policy, Rutgers The State University of New Jersey New Brunswick New Jersey USA

**Keywords:** COVID‐19, grocery shopping, online grocery shopping, retail and wholesale trade; e‐commerce

## Abstract

The COVID‐19 pandemic altered daily activities. Many consumers reverted to online grocery shopping and home delivery. We analyze factors associated with the decision to grocery shop online and whether this will persist post‐COVID using data collected via a representative online Qualtrics panel in the State of New Jersey (*N* = 1,419). Around half of respondents either decreased in‐person shopping, increased online shopping, or pursued a combination of both. We used factor analysis to decompose attitudes towards the pandemic, finding that attitudinal responses broke down into ‘fearful’, ‘believers’, and ‘deniers’. Binomial regressions were used to analyze patterns of frequency of grocery shopping during the pandemic and changes in behavior during the pandemic. Results suggest that age, gender, ethnicity, educational attainment, having children at home, and attitudes towards COVID‐19 are likely to influence frequency of online and in‐person grocery shopping. Specifically, being 50 years or older is negatively associated with online grocery shopping. Those who deny COVID‐19 were less likely to decrease in‐person grocery shopping. People who had children at home, who had advanced degrees, or who were of Hispanic origin were more likely to increase online shopping and decrease in‐person shopping during the pandemic. While our results suggest that in‐person grocery shopping will return to prepandemic levels, we found that respondents report some increased persistence in online grocery shopping post‐COVID.

## 1 INTRODUCTION

Shopping for groceries is an integral part of people's lives. During the early stages of the pandemic, prior to and during the implementation of ‘stay‐at‐home’ orders, this activity was severely disrupted. Grocery shelves were empty because of hoarding, and in some cases, supply disruptions. Many turned to online grocery shopping as an alternative, but at the time, the ability to book a delivery slot online was almost impossible, as demand outstripped the ability of online grocery delivery companies to provide service, although this eventually was resolved, allowing those who desired to purchase groceries online to do so. In this study we analyze representative data collected in New Jersey to determine what changes in grocery shopping habits might persist after the pandemic subsides.

From 2019 to 2020, US food‐at‐home expenditure increased from $808.0 billion to $876.8 billion. Meanwhile, food‐away‐from‐home spending (such as take‐out, restaurant pick‐up, or eating in a restaurant) decreased from $978.2 billion to $813.4 billion. For the first time since 2008, grocery food expenditures accounted for more than 50% of total food expenditures (USDA, 2021). Needless to say, the COVID‐19 pandemic has transformed the way people shop for groceries. In 2019, around 11% of people ordered groceries online at least once a month. In 2021, that number jumped to 23%. On the other hand, frequent in‐person grocery shopping (more than once a week) declined. People are buying more food and making fewer trips to the grocery store, are shopping more frequently online, or both (Brenan, 2021). The type of food ordered online has changed as well. Fresh foods purchased online increased by 200% during the pandemic, and began to slow down mid‐2021 (Dillard, 2021).

Prior to the pandemic, online grocery shopping made up a small but growing share of the market. The proportion of online shoppers surged, with 45% of consumers reporting shopping online for groceries more now than before the pandemic. When normalcy returns, the expectation is that some people will continue to shop for groceries online.

Our analysis is based on a survey with 1,419 respondents in New Jersey, collected via an online Qualtrics panel. The survey included a variety of questions to measure transportation behavior before and during the pandemic, and expectations of change after the pandemic. Among these were questions aimed at examining grocery shopping behavior before, during, and after (expected) the COVID‐19 pandemic. The survey was administered between November 2020 and February 2021 (coincidentally the first winter peak of the pandemic in New Jersey).

Our study contributes to the literature by analyzing the factors associated with shopping for groceries online before, during, and expected post‐pandemic; shopping for groceries in‐person before, during, and post‐pandemic; and changes in grocery shopping behavior due to the pandemic. We also explore the factors that increase or decrease willingness to grocery shop online after we return to normalcy using New Jersey as a case study. We include sociodemographic and economic variables previously considered in the online grocery shopping literature, while also including the following novel determinants: those who have the availability of working from home, attitudes towards the COVID‐19 pandemic (deemed as ‘believers’, ‘deniers’, and ‘fearful’), and frequency of in‐person and online shopping before the pandemic.

## 2 LITERATURE REVIEW

We used the Web of Science (WOS) database to gather literature on online grocery shopping. Our search string included ‘online grocery shopping’, ‘online food shopping’, and ‘e‐grocery shopping’, and yielded 115 articles, 62 (54%) of which were published in 2020 or 2021. Sixteen (14%) of the articles focus on the impact of the COVID‐19 pandemic on online grocery shopping.

### 2.1 Studies of online grocery shopping before COVID‐19

Online grocery shopping has had a small but growing share of the grocery shopping market in the United States. In 2019, it was estimated that 43% of Americans had purchased groceries online, 21% did so monthly, and 10% biweekly (Cohen et al., 2020; Jones & Kashanchi, 2019). Nonetheless, even those who are regular users of online grocery shopping services still shopped in‐person at least some of the time (Pitts et al., 2018). Online grocery shopping is more common among those with children and upper‐income adults earning more than $100,000. Adults employed full time were slightly more likely to buy groceries online. Additional reasons for online grocery shopping prior to the pandemic include avoiding crowds and long lines, caring for a sick family member, or transitioning to a new residence (Pitts et al., 2018). Barriers to online grocery shopping included the inconvenience of waiting for deliveries, delivery fees, out of stock items, and inappropriate substitutions. Eighty‐three percent of Americans shopped in‐person for groceries at least once a week before the pandemic, and this declined slightly to 79% in 2021 (Brenan, 2021).

Various demographic factors have been found to be associated with the likelihood of engaging in online grocery shopping. Generally, younger adults, males, having children, high educational attainment, and being employed have a positive influence on online grocery shopping behavior (Farag et al., 2007; Goethals et al., 2012; Hiser et al., 1999; Jaller & Pahwa, 2020; Naseri & Elliott, 2011; Van Droogenbroeck & Van Hove, 2017). Higher income levels have been found to have a positive effect on online grocery shopping behavior (Hansen, 2005; Hui & Wan, 2009) or an insignificant one (Hiser et al., 1999; Naseri & Elliott, 2011). Van Droogenbroeck and Van Hove (2017) surveyed Belgian shoppers and found that, after controlling for both personal‐ and household‐level characteristics, being aged 31–50, having a college degree, presence of young children, and employed adults were found to be positively associated with online grocery shopping. Gender was insignificant, and income was not included in their model.

### 2.2 Studies focusing on the impact of COVID‐19

A growing body of online grocery shopping studies focuses on the COVID‐19 pandemic. Grashuis et al. (2020) found that consumer expenditures on groceries during the pandemic increased, and consumers were less willing to shop inside a store when COVID‐19 was spreading at an increasing rate. Kim (2020) suggested that the success of online grocery delivery apps shows that consumers are willing to purchase groceries online if post‐purchase customer service is ensured (Kim, 2020). Baarsma and Groenewegen (2021) studied the effect of the pandemic on demand for online grocery shopping in the Netherlands. They found that, for each additional hospital admission, traffic on an online grocery shopping app increased by 7.3% and sales per order by 0.31% (Baarsma & Groenewegen, 2021). Similarly, another study found that an additional confirmed case of COVID‐19 increased e‐commerce sales by 5.7% in Taiwan (Chang & Meyerhoefer, 2021). In a May 2020 survey of two US cities, Chenarides et al. (2021) found that three‐fourths of the respondents were not buying the food they preferred owing to stock limitations. People were, however, buying more groceries than normal. They found a 255% increase and a 158% increase in pick‐up and delivery grocery services, respectively (Chenarides et al., 2021).

Shamim et al. (2021) found in an online survey that females were more likely to pursue health safety practices during grocery shopping. People reduced their frequency of grocery shopping and tried to shop quickly and efficiently (Shamim et al., 2021). Another study found that people with higher educational attainment were more likely to buy groceries online, which is in line with prepandemic behavior (Alaimo et al., 2020). Ferrante et al. (2021) administered a survey to US parents with children 4–8 years old in October 2020. Forty‐nine percent of the parents reported increases in use of online grocery shopping. Meanwhile, 48% reported going to the grocery store less than they did prior to the pandemic, and 26% indicated increasing frequency (Ferrante et al., 2021). Adoption of online shopping during the pandemic was negatively associated with age (aged 65+) (Wang et al., 2021). Jensen et al. (2021) analyzed online grocery shopping decisions and frequency during and expectations after the pandemic in a survey of 1,558 US households. They found that 55% of respondents shopped online in June 2020, and 20% were first timers. Younger adults, males, people who have children, and people who are employed full time were more likely to online shop at least once during the pandemic than other groups. Income and educational attainment were not associated with decisions to online shop. People who had not online grocery shopped in the past were less likely to online grocery shop during the pandemic. They also found that shopping in‐person during the pandemic was negatively associated with the decision to online shop. People who were concerned with becoming ill were more likely to shop online but there was no significant impact on the frequency of online shopping. They found that 58% of respondents planned to continue online grocery shopping (Jensen et al., 2021).

## 3 DATA, DESCRIPTIVE STATISTICS, AND METHODS

### 3.1 Description of New Jersey

New Jersey is situated along the Northeast Corridor between the states of Pennsylvania and New York. The state is anchored by the large metropolitan areas of Philadelphia in the west and New York City in the northeast, leading to large commuter flows to both cities. New Jersey includes large cities adjacent to New York, including Jersey City, opposite New York City across the Hudson River, and Newark, a few miles inland along the Passaic River. The state itself is highly urbanized, with a population of about 8.9 million, but contains large areas with rural landscapes and many communities along the Eastern Seaboard, which attract seasonal populations (Chapman et al., 2021). We summarize New Jersey demographics in Table 1.

**TABLE 1:** Sample demographics relative to NJ census totals (2019 ACS 5‐year estimates)

Variable	*n*	Sample (%)	New Jersey (%)
**Age:**			
18–24	203	14.3	11.5
25–34	305	21.5	17.0
35–44	303	21.4	17.0
45–54	267	18.8	18.8
55–64	233	16.4	17.9
65 and over	108	7.6	18.1
**Gender:**			
Male	605	42.6	48.9
Female	803	56.6	51.1
Other	5	0.4	N/A
Prefer not to answer	6	0.4	N/A
**Income:**			
Under $49,999	434	30.6	31.5
$50,000 to $74,999	246	17.4	14.6
$75,000 to $99,999	213	15.0	12.1
$100,000 to $149,000	284	20.0	17.6
$150,000 to $199,999	132	9.3	10.1
$200,000 or more	109	7.7	14.0
**Education:** ^a^			
High school or less	171	15	26.9
Some college but no degree	188	15.5	15.7
Associate's	115	9.4	6.4
Bachelor's	386	31.7	25.1
Master's or doctoral degree	356	29.3	16.1
**Race:**			
White/Caucasian	889	62.7	71.9
Other	72	5.1	N/A
Asian	118	8.3	10.0
Two or more races	57	4.0	2.3
Black/African American	246	17.3	15.1
Prefer not to answer	22	1.6	N/A
American Indian or Alaska native	13	0.9	0.6
Native Hawaiian or Pacific Islander	2	0.1	0.1
**Hispanic:**			
No	1,110	78.3	79.1
Prefer not to answer	8	0.6	N/A
Yes	300	21.2	20.9
Sample size	1,419		

^a^ Educational attainment is measured for the population 25 years and older, *N* = 1,216.

### 3.2 Description of survey

A survey was designed to capture a variety of changes in transportation‐related behavior due to the COVID‐19 pandemic. Expectations for how travel activity would adjust post‐pandemic were also obtained. The survey contained a variety of attitudinal and demographic questions, as well as those focused on travel behavior; our analysis here focused on grocery shopping behavior. The data were collected via a Qualtrics online panel 1 and were set to be representative of New Jersey residents (Table 1). The survey was deployed between November 2020 and February 2021, which coincided with the first winter peak in COVID‐19 cases, hospitalizations, and deaths in the United States (*The New York Times*, 2021). The final sample contains 1,419 complete responses. Table 1 presents the sample demographics relative to the New Jersey census (2019 ACS 5‐year estimates). Our sample is representative of New Jersey relative to income, gender, age, and race and is skewed towards highly educated individuals.

Our use of an online panel for data collection has some limitations. The respondents receive compensation for their participation in the survey; this may include small cash payments, airline points, and credits towards gift cards. We rely on Qualtrics to guarantee a representative sample for our target population; however, there could be residual biases in the data. The main one is that the survey is conducted online, whether on a desktop or a mobile phone, so those without access to the Internet would be excluded. Paid participants may also not be as thoughtful in filling out survey questions; however, Qualtrics uses various algorithms to check this, such as eliminating respondents who do the survey too quickly and who, for example, select the first option for every question, among other checking techniques.

### 3.3 In‐person grocery shopping and online grocery shopping

**FIGURE 1:**
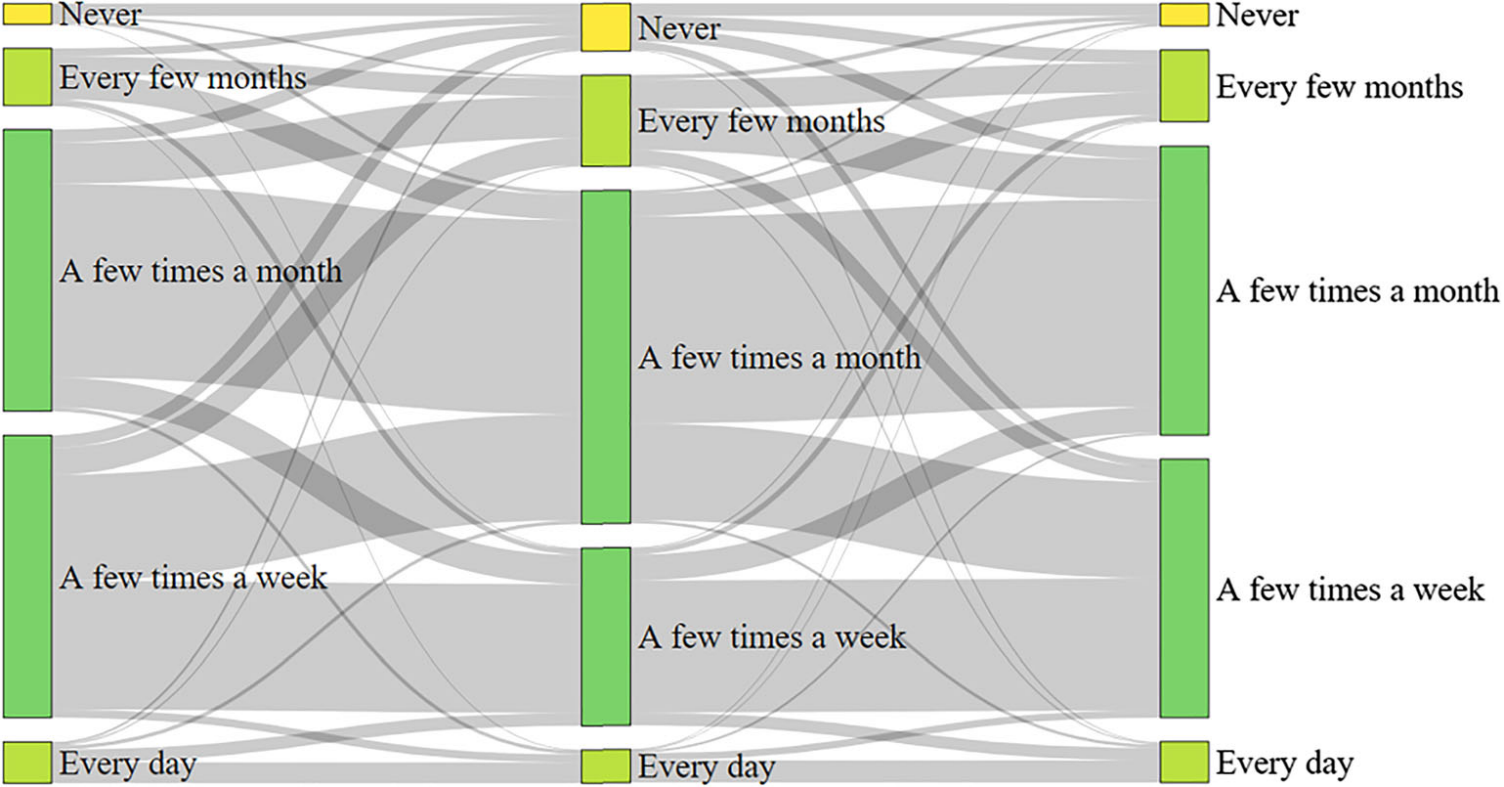
Sankey diagram of in‐person grocery shopping frequency before, during, and after the pandemic

**FIGURE 2:**
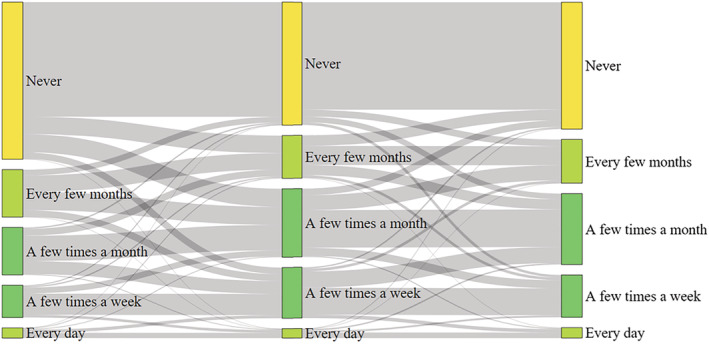
Sankey diagram of online grocery shopping frequency before, during, and after the pandemic

Data were gathered on the frequency of shopping for groceries online and in‐person before and during the pandemic, and in the future (Table 2). Generally, people decreased in‐person grocery shopping and increased online grocery shopping. Based on respondent answers, in‐person grocery shopping is expected to return to prepandemic levels in the future, while online grocery shopping is expected to persist (Figures 1 and 2). About 89% of individuals shopped for groceries in‐person at least a few times a month before the pandemic began, which decreased to 80% during the pandemic. In‐person grocery shopping is expected to return to near prepandemic levels after the pandemic ends (86%). About 30% of respondents shopped for groceries online at least once a month before the pandemic. This jumped to 44% during the pandemic and is expected to stay near that level (42%) after the pandemic ends. About 53% (756) of respondents had never used online grocery shopping services before the pandemic began. One‐quarter (27%; 205) of those who had never previously shopped for groceries online did so for the first time during the pandemic, and 64% of these were new to online grocery shopping (132) and planned to continue online grocery shopping once the pandemic subsides.

**TABLE 2:** Responses for grocery shopping behavior before, during, and expected after the pandemic

	Before pandemic		During pandemic		In the future	
	*n*	(%)	*n*	(%)	*n*	(%)
**Shop for groceries in a store:**						
Never	43	3.03%	99	6.98%	47	3.31%
Every few months	119	8.39%	189	13.30%	149	10.50%
A few times a month	584	41.20%	693	48.80%	600	42.30%
A few times a week	587	41.40%	369	26.00%	537	37.90%
Every day	86	6.06%	69	4.86%	85	5.99%
**Shop for groceries online for delivery:**						
Never	756	53.40%	592	41.70%	611	43.10%
Every few months	228	16.10%	207	14.60%	211	14.90%
A few times a month	228	16.10%	329	23.20%	342	24.10%
A few times a week	155	10.90%	245	17.30%	204	14.40%
Every day	49	3.46%	46	3.24%	50	3.53%

During the pandemic, people were urged to stay home as much as possible; hence, we were interested in how many respondents decreased in‐person shopping behavior, increased online shopping, or did both (Table 3). We found that 47% either increased online shopping (191), decreased in‐person shopping (260), or did both (214). Fifty‐three percent either made no change to their grocery shopping practices, or made minor changes in frequency that could not be captured by our survey.

**TABLE 3:** Change in grocery shopping behavior during the pandemic

	No measurable increase in online grocery shopping	Increase in online grocery shopping
**No measurable decrease in in‐person grocery shopping**	751 (53%)	191 (13.5%)
**Decrease in in‐person grocery shopping**	260 (18.4%)	214 (15.1%)

### 3.4 Factor analysis to decompose attitudinal questions

An exploratory factor analysis (EFA) was conducted to decompose attitudes toward the pandemic. Respondents were presented with a set of questions to measure their attitudes toward the COVID‐19 pandemic and measures taken against it (Table 4). A five‐point Likert scale from ‘strongly disagree’ to ‘strongly agree’ was used in the survey. Factor analysis provides a way to simplify these types of attitudinal questions on the basis of their correlations and allows a subjective interpretation of those attitudes (Harman & Jones, 1966; Revelle, 2009). On the basis of an examination of the data, we found that three factors loaded well on the questions. We performed an orthogonal rotation, and defined attitudes as those ‘fearful’ of COVID, ‘believers’ in COVID, and ‘deniers’ that COVID is a threat. These factor loadings are used as independent variables in our multivariate analyses. The factor loadings bolded in Table 4 indicate the questions that load on each factor.

**TABLE 4:** Attitudinal questions from survey and factor loadings

Attitudinal questions from survey	Believers	Deniers	Fearfuls
I am concerned that if I catch the coronavirus, I will be very ill.	0.22	0.06	**0.65**
I am concerned that friends or family members will be very ill if they catch the coronavirus.	−0.04	−0.03	**0.97**
Everyone should just stay home as much as possible until the coronavirus has subsided.	**0.94**	−0.06	0.01
Society is overreacting to the coronavirus.	0.00	**0.99**	−0.03
Shutting down businesses to prevent the spread of coronavirus is not worth the economic damage that will result.	−0.06	**0.67**	0.08
My friends and family expect me to stay at home until the coronavirus subsides.	**0.57**	0.19	0.12
Everyone should wear a face mask when they are near other people.	**0.41**	−0.17	0.34

### 3.5 Regression analysis for grocery shopping during the pandemic

The respondents were asked about how often they shopped for groceries in‐person and online for delivery before, during, and expected after the pandemic is no longer a concern. We estimate a binomial regression for the two shopping behaviors (in‐person versus online) during these three periods for a total of six binary regressions (full results in the Supplementary Material). The models provide insight on how grocery shopping is shaped by different demographic and attitudinal behaviors. In general, we hypothesize that younger people, those with children, and more affluent households are more likely to shop for groceries online, as are those who are more fearful of COVID‐19; we have no pre‐existing hypotheses on most other demographic variables.

We define the two variables as follows: (1) frequent in‐person grocery shoppers are those who shop for groceries in a brick‐and‐mortar store a few times a week or more, and (2) frequent online grocery shoppers are those who use delivery services a few times a month or more. The definition of ‘frequent’ for online and in‐person shopping differs, because it is relative to how frequent each shopping activity is. In‐person grocery shopping is far more frequent than online grocery shopping, and even frequent users of online grocery shopping services usually make in‐person grocery trips on a regular basis. The percentages of ‘frequent shoppers’ for each category and each time interval are shown in Table 5. The independent variables used in the models are gender, presence of children in the household, age, income, education, race/ethnicity, number of vehicles available per person, employment type, frequency of shopping prior to the pandemic, and attitudes toward the pandemic.

**TABLE 5:** Definition of dependent variable for binary logit models

Dependent variable: Frequent shopper definition	Before	During	After (expected)
Shop for groceries in a store at least a few times a week	47.5%	30.9%	43.9%
Shop for groceries online for delivery at least a few times a month	30.5%	43.7%	42.0%

### 3.6 Regression analysis for changes in grocery shopping behavior during the pandemic

Most of our respondents reported no changes in their grocery shopping behavior during the pandemic. About 29% of our respondents reported increasing online grocery shopping, 33% reported decreasing in‐person grocery shopping, and 15% did both (Table 3). Two binomial logit models were estimated to analyze the change in the two types of grocery shopping behaviors. The first model estimates decreases in the frequency of in‐person grocery shopping (yes, 471; no, 931) and the second model estimates increased frequency of online grocery shopping (yes, 399; no, 1,000) after removing incomplete observations). The models control for sociodemographic variables, work‐from‐home status, attitudes towards the COVID‐19 pandemic, and previous history of grocery shopping before the pandemic.

## 4 RESULTS

### 4.1 Frequency of in‐person and online grocery shopping during the pandemic

Table 6 displays results for binomial logit models of the frequency of both in‐person grocery shopping and online grocery shopping during the pandemic. 2 Our independent variables include respondent demographics and socioeconomics, plus our factor loadings for attitudinal questions.

**TABLE 6:** Frequent in‐person and online grocery shoppers during the pandemic

	*Dependent variable:*			
	Frequent in‐person grocery shopping (weekly) during the COVID‐19 pandemic (1) (yes, 438; no, 981)		Frequent online grocery shopping (monthly) during the COVID‐19 pandemic (2) (yes, 432; no, 984)	
	Coef. (SE)	Odds ratio	Coef. (SE)	Odds ratio
Female (ref.: male)	**−0.219** * **(0.130)**	**0.803**	0.060 (0.148)	1.062
Children present	−0.085 (0.143)	0.919	**0.467** **(0.156)**	**1.595**
**Education (ref.: high school or less)**				
Associates	0.184 (0.235)	1.202	**0.440** * **(0.256)**	**1.553**
Bachelor's	**0.433** ** **(0.174)**	**1.541**	**0.397** ** **(0.192)**	**1.488**
Master's	**0.579** *** **(0.200)**	**1.784**	0.311 (0.225)	1.365
Doctoral degree	0.306 (0.411)	1.358	**1.260** ** **(0.537)**	**3.527**
Professional degree	0.426 (0.402)	1.531	0.652 (0.451)	1.918
**Income (ref.: <$35,000)**				
35,000–49,999	−0.141 (0.277)	0.868	0.290 (0.302)	1.336
50,000–74,999	−0.091 (0.217)	0.913	0.137 (0.233)	1.147
75,000–99,999	−0.009 (0.227)	0.991	0.264 (0.246)	1.302
100,000–124,999	0.092 (0.252)	1.097	0.264 (0.280)	1.303
125,000–149,999	0.395 (0.263)	1.484	**0.914** *** **(0.314)**	**2.495**
>150,000	0.063 (0.235)	1.065	0.386 (0.261)	1.471
**Age group, years (ref.: 18–24)**				
25–34	0.0004 (0.231)	1.000	0.110 (0.240)	1.116
35–49	−0.017 (0.236)	0.983	−0.109 (0.248)	0.897
50–64	0.321 (0.241)	1,378	−0.376 (0.258)	0.687
>65	0.228 (0.320)	1.256	−0.386 (0.364)	0.680
**Race**				
Black	0.146 (0.173)	1.157	0.299 (0.189)	1.349
Asian	−0.057 (0.230)	0.945	0.145 (0.252)	1.156
Hispanic/Latino	0.178 (0.166)	1.195	0.232 (0.183)	1.261
**Transportation**				
Vehicles per person (continuous)	0.029 (0.135)	1.030	**−0.420** ***, ^b^ **(0.155)**	**0.657**
**Employment type (ref.: Can work from home)**				
Cannot work from home	−0.066 (0.151)	0.936	**−0.599** *** **(0.170)**	**0.550**
Not employed	**−0.376** * **(0.196)**	**0.687**	**−0.451** ** **(0.211)**	**0.637**
**Attitude towards COVID‐19**				
Believers	0.136 (0.117)	1.145	**0.283** ** **(0.135)**	**1.327**
Deniers	**0.534** *** **(0.073)**	**1.706**	**0.203** ** **(0.086)**	**1.226**
Fearful	−0.065 (0.112)	0.937	**0.216** * **(0.129)**	**1.242**
**Previous shopping experience**				
Never shopped online before			**−2.403** *** **(0.152)**	**0.090**
Constant	**−1.096** *** **(0.299)**		**0.718** ** **(0.325)**	
Observations	1,391		1,391	
Log likelihood	−754.244		−748.133	
Akaike Information Criterion	1,550.487		1,538.266	
TJUR2	0.19		0.12	

*Note*: Number in parentheses represents the standard error.

* *p* < 0.10;

** *p* < 0.05;

*** *p* < 0.01.

^b^ Even if we exclude the ‘never shopped online before’ variable and have all the variables be the same for comparison purposes, the coefficient for vehicles per person (−0.334) is still higher than before the pandemic (−0.301).

Respondents who identified as female were less likely to grocery shop in‐person during the pandemic than their male counterparts. Having children present in the home was positively associated with grocery shopping in‐person before the pandemic, but not during or expected after. Educational attainment was generally positively associated with frequent in‐person grocery shopping before and during the pandemic. People aged 50+ were associated with more in‐person shopping before the pandemic but not during or expected after the pandemic. Those who are unemployed were less likely to decrease in‐person grocery shopping during the pandemic. Race/ethnicity, income, and vehicles available per person were not associated with differences in in‐person shopping before, during, or expected after the pandemic. Those classified as ‘deniers’ by our factor analysis were more likely to shop in‐person during the pandemic.

Male respondents were more likely to shop for groceries online before the pandemic, but not during or expected after. People who have children present at home are more likely to online grocery shop for delivery before and during the pandemic. A positive, but weak, association between education and online grocery shopping was observed during the three time periods. No meaningful difference in behavior based on income was detected. Those aged 50+ are less likely to order groceries online than younger groups before but not during the pandemic. People of Black and/or Hispanic/Latino origin were more likely to report online grocery shopping at least once a month than other groups after the pandemic. Asians were less likely to online grocery shop before the pandemic but not during or after. The number of vehicles per person was negatively associated with online grocery shopping, meaning that those with no cars were more likely to online shop than those with cars available. People who had a job that allowed them to work from home at least some of the time were more likely to online grocery shop for delivery than those who have a job that does not allow them to work from home or who were unemployed.

‘Fearfuls’ and ‘believers’ of COVID‐19 were more likely to online shop during the pandemic, but not before or after. Those deemed as ‘deniers’ of COVID‐19 were more likely to online grocery shop for delivery at least once a month before, during, and after the pandemic. However, the magnitude of the coefficient decreases during the pandemic.

### 4.2 Changes in grocery shopping behavior from before the pandemic

The results of the models estimating the change in grocery shopping behavior from before to during the pandemic are displayed in Table 7. Generally, we find no associations with demographic and socioeconomic measures with decreases in in‐person shopping, except for those with children at home. Those who cannot work from home or are not employed were more likely to decrease in‐person grocery shopping. Those who we classify as COVID ‘deniers’ on the basis of our attitudinal factors are more likely to shop in‐person for groceries. We also included control variables for previous grocery shopping behavior. Those who were more frequent (weekly) in‐person grocery shoppers were more likely to decrease their in‐person shopping, while the opposite was true for frequent online grocery shoppers. Those who had never shopped online for groceries also increased their in‐person grocery shopping relative to those who had shopped online previously.

**TABLE 7:** Binomial regression results for changes in grocery shopping behavior

	*Dependent variable:*			
	Decrease in in‐person shopping (1) (yes, 471; no, 931)		Increase in online shopping (2) (yes, 399; no, 1,000)	
	Coef. (SE)	Odds ratio	Coef. (SE)	Odds ratio
Female (ref: male)	0.206 (0.137)	1.229	0.073 (0.138)	1.076
Children present	**0.291** ** **(0.145)**	**1.338**	**0.263** * **(0.143)**	**1.301**
**Education (ref.: high school or less)**				
Associate's	−0.232 (0.238)	0.793	0.266 (0.237)	1.305
Bachelor's	−0.027 (0.171)	0.973	**0.477** *** **(0.174)**	**1.611**
Master's	−0.082 (0.194)	0.921	0.278 (0.199)	1.320
Doctoral degree	0.500 (0.417)	1.648	0.524 (0.412)	1.688
Professional degree	−0.213 (0.424)	0.808	0.380 (0.415)	1.463
**Age**				
50 years old or older	−0.105 (0.161)	0.901	**−0.556** *** **(0.164)**	**0.574**
**Race**				
Black	0.004 (0.171)	1.004	0.104 (0.174)	1.110
Asian	0.074 (0.233)	1.076	0.160 (0.224)	1.173
Hispanic/Latino	0.205 (0.165)	1.228	**0.273** * **(0.163)**	**1.314**
**Transportation**				
Vehicles per person (continuous)	−0.080 (0.144)	0.923	−0.091 (0.143)	0.913
**Employment type (ref: Can work from home)**				
Cannot work from home	**0.294** * **(0.168)**	**1.342**	**−0.431** *** **(0.162)**	**0.650**
Not employed	**0.702** *** **(0.199)**	**2.017**	−0.283 (0.198)	0.753
**Attitude towards COVID‐19**				
Believers	−0.017 (0.126)	0.983	−0.058 (0.127)	0.943
Deniers	**−0.322** *** **(0.080)**	**0.724**	−0.117 (0.081)	0.890
Fearful	0.110 (0.119)	1.117	**0.213** * **(0.121)**	**1.237**
**Previous shopping experience**				
Frequent in‐person shopping (≥1 per week)	**1.857** *** **(0.138)**	**6.406**	**0.533** *** **(0.133)**	**1.704**
Frequent online shopper (≥1 per month)	**−0.563** *** **(0.190)**	**0.569**	**−1.942** *** **(0.196)**	**0.143**
Never shopped online before	**−0.727** *** **(0.171)**	**0.483**	**−1.106** *** **(0.158)**	**0.331**
Constant	**−1.669** *** **(0.279)**		−0.142 (0.268)	
Observations	1,391		1,391	
Log likelihood	−754.244		−748.133	
Akaike Inf. Crit.	1,550.487		1,538.266	
TJUR2	0.19		0.12	

*Note*: Number in parentheses represents the standard error.

* *p* < 0.10;

** *p* < 0.05;

*** *p* < 0.01.

People who increased online grocery shopping also tended to have children present in the household, be infrequent online grocery shoppers before the pandemic, be frequent in‐person grocery shoppers, and have a job that allows them to work from home. For those who increased their online grocery shopping, some associations are present with demographic and socioeconomic indicators. Specifically, those with Hispanic/Latino heritage and those with a bachelor's degree are more likely to increase online grocery shopping, though there is no obvious trend in educational level. Those over the age of 50 are much less likely to increase their online grocery shopping, suggesting a potential divide in ability to access groceries safely.

### 4.3 The future of online grocery shopping

On the basis of our survey, online grocery shopping looks set to see a permanent increase post‐pandemic. Relative levels of online grocery shopping, based on respondent expectations, will be similar in the future as during the pandemic. Sixty‐four percent of those who shopped for groceries online for the first time expect to continue online grocery shopping at least every few months after the pandemic subsides.

We were interested in the reasons behind changes in online grocery shopping behaviors. Almost one‐third of respondents (29%) reported that they would online grocery shop more for groceries once the pandemic subsides than they did before the pandemic began. Respondents who reported that they would increase their online grocery shopping were asked why they would continue to do so. The top three reasons for this sustained shift in behavior were: to save time, to avoid trips to the store, and to be able to shop 24/7 (Table 8).

**TABLE 8:** Survey question for reasons for increasing online shopping post COVID

You told us that you expect to shop for groceries online more when COVID‐19 is no longer a threat than you did pre‐COVID. Why? (*N* = 356)	*n*	%	Male (%)	Female (%)	Age 18–24 (%)	Age 25–54 (%)	Age 55+ (%)
	356		45.5	52.8	19.1	64.5	16.4
It saves time	220	53.92%	45.0	54.5	13.6	67.4	19.0
It's easier to stick to my shopping list	131	32.11%	42.7	55.7	16	71.1	12.9
I do not have to carry my groceries	124	30.39%	37.9	60.5	19.3	60.6	20.3
I can shop 24/7	155	37.99%	38.1	60.0	20.0	64.0	16.0
I can more easily compare prices	101	24.75%	41.6	55.4	23.8	71.2	5.0
I can avoid going to stores	161	39.46%	42.2	56.5	15.5	64.6	19.9
I have a wider variety of choices	57	13.97%	42.1	54.4	21.1	63.2	15.8
Other (please specify):	13	3.19%					

We performed cross‐tabulations with the answers to the questions in Table 8 across age and gender. Compared with male respondents, female respondents were more likely to prefer not having to carry their groceries as a reason for online grocery shopping. Those aged 18–24 liked being able to easily compare prices and were less sensitive to time savings than other age groups. Compared with other age groups, those aged 55 and older were more likely to like online grocery shopping owing to not having to carry their own groceries, avoid going to the stores, and saving time.

On the other hand, the three top‐reported reasons for reducing online grocery shopping after the COVID‐19 pandemic reported by our respondents were: concerns that groceries would not be delivered fresh, not wanting to wait for groceries to arrive, and that the product information online is not always accurate (Table 9). We do not report cross‐tabulations for this question as the sample size was too small.

**TABLE 9:** Survey question for reasons for decreasing online shopping post COVID

You told us that you plan to shop for groceries online less when COVID‐19 is no longer a threat than you did pre‐COVID. Why? (*N* = 127)	*n*	%
I cannot sample or inspect what I'm buying	35	25.93%
I do not always get what I ordered	33	24.44%
I have difficulty using the online shopping apps or website	34	25.19%
Product information online is not always accurate	39	28.89%
I am concerned that my groceries will not be fresh	47	34.81%
I do not want to wait for my groceries to arrive	40	29.63%
I do not want to pay for delivery	34	25.19%
I am concerned about privacy or security in online shopping	19	14.07%
Other (please specify):	5	3.70%

## 5 DISCUSSION

In this study, we sought to understand how grocery shopping behavior changed during the pandemic and potentially in the future. In‐person grocery shopping is generally not influenced by sociodemographic variables in the same way that online grocery shopping is, since the vast majority of people grocery shop in‐person at least once a month. During the pandemic, males were slightly more likely to shop in‐person for groceries than females. Moreover, we did find that changes in behavior were associated with several characteristics. People who had children at home, who had a job that did not allow them to work from home, or who were unemployed, were significantly more likely to decrease in‐person grocery shopping during the pandemic. On the other hand, ‘deniers’ of the pandemic were significantly less likely to decrease in person grocery shopping, an unexpected finding that we elaborate on below.

For those who continued to shop in‐person for groceries, there are various strategies to reduce potential risk from COVID. Some may shop less frequently but purchase larger quantities of food; others may shop quickly, but purchase less. Some may have shifted when they shopped to times when stores were less crowded, such as early morning; many stores reserved this time slot for older and immunocompromised customers. Our survey was not designed to capture the details of how people shop; merely their reported frequency of shopping, whether in person or online.

Online grocery shopping remains a small share of the grocery shopping market, although our survey (and multiple national surveys) indicates growth (Brenan, 2021). We found that before the pandemic, males, having children at home, and younger adults (<50), were more likely to online grocery shop, which is in line with previous literature (see Supplementary Material for full model results). Income and education were interestingly not statistically significant for online grocery shopping prior to the pandemic, which some previous studies have found as well (Hiser et al., 1999; Naseri & Elliott, 2011). We found that lower car ownership in a household is associated with more online grocery shopping, both before and during the pandemic, a finding not reported in prior studies. This suggests that the ability to shop online can provide some carless households with more access to fresh foods.

Previous studies have analyzed factors associated with the propensity to online grocery shop during the pandemic, without specifically looking at the changes in frequency, as we do in this study. The main drivers of changes in grocery shopping behavior stemmed from (1) having children at home, (2) being able to work from home, (3) attitudes towards COVID‐19, and (4) previous grocery shopping behavior before the pandemic began.

One of our main contributions is to decompose attitudes towards COVID‐19 and include these in our models. One previous study found the variable ‘concerned with becoming very ill’ as significant in online shopping, which is similar to what we found since ‘fearfuls’ were correlated with that question (Jensen et al., 2021). ‘Deniers’, or those who believed that society was overreacting to the pandemic and that the economy is suffering unnecessarily, had an interesting relationship with grocery shopping that had not been considered in other studies. ‘Deniers’ were less likely to decrease in‐person grocery shopping during the pandemic and ‘fearfuls’ were more likely to increase online grocery shopping. ‘Believers’ was not a significant variable in the changes in grocery shopping behavior; however, it was a significant variable in being a frequent online grocery shopper during the pandemic. While our survey did not measure political beliefs, it is likely that the ‘deniers’ were more likely to have more conservative values and beliefs. We can speculate that those who are ‘deniers’ increased their in‐person grocery shopping as an act of defiance of the pandemic, with potential implications for viral spread (Noland, 2021).

An important finding is that those over the age of 50 are less likely to shop for groceries online; this may be due to unfamiliarity with the technology or systems available to access online grocery shopping. In any case, this finding suggests a need to find ways to make online grocery shopping easier and/or more accessible for older population groups. Older people are more vulnerable to COVID‐19, and this group was far less likely to shop online for groceries at least once a month before the pandemic than younger people (<50). During the pandemic, our age variable became insignificant after including the variable ‘Never online grocery shopped before the pandemic’. When controlling for that variable, older people are not any less likely to grocery shop online; however, we know from the data that a much smaller proportion of older people (12%), online grocery shopped before the pandemic than people under the age of 50 (40%). Thirty‐nine percent of respondents aged 65 or older compared with 31% of those aged 18–24 decreased in‐person grocery shopping; a difference not statistically significant in our model.

As for the long‐term impacts of the pandemic on grocery shopping, the results of our survey suggest that in‐person grocery shopping will return to normal prepandemic levels, while online grocery shopping will continue to persist. The top reason for continuing online grocery shopping was time savings (Table 8), while those who plan to shop online for groceries less were generally concerned with product freshness, the reliability of orders, and not being able to inspect their purchases (Table 9). People who hold a master's degree or PhD, people who are African American or of Hispanic origin, people who have a job that allows them to work from home, and people who earn at least $125,000 per year are more likely to continue to online grocery shop after the pandemic subsides. About 64% of those who online grocery shopped for the first time during the pandemic reported they would continue to do so after the pandemic subsides. From the supply side, it might be beneficial for grocers and online delivery apps to continue to add curbside pick‐up and delivery options for those who prefer to purchase groceries online in the long run.

Working‐from‐home status proved to be a more significant variable in all of our online grocery shopping and change in grocery shopping models than ‘essential worker’. A worker may be considered essential and still be able to work from home (i.e., a construction project engineer who does mostly office work, or a teacher who teaches classes online during the pandemic). The distinction of being able to work from home is more important, particularly in the context of online grocery shopping, since those who cannot work from home are less likely to online grocery shop before, during, and after the pandemic. Previous studies only analyzed employment type as full time, part time, or unemployed, rather than whether employees were essential or worked at home (Jensen et al., 2021; Pitts et al., 2018).

## 6 CONCLUSIONS

Our survey was conducted in November 2020, which coincided with the height of the pandemic in the United States and New Jersey. Over a year later, as of this writing, many people continue to work from home, the Omicron surge increased cases and death rates over the winter months, and day‐to‐day life is still very different from what it was before the pandemic began. Future research should continue to account for COVID‐related life changes, such as working from home, attitudes toward the pandemic, and previous and current shopping behavior. The exogenous shock to habitual behaviors, and how or if people change, will be a rich area for further study.

Our findings do not suggest that online grocery shopping has a meaningful impact on the transport system, at least not at the consumer level. Goods from online grocery shopping must still be delivered by the grocer or a third party, which may slightly decrease emissions depending on the circumstances and number of deliveries per trip. If picked up via curb side, the vehicle emissions are the same as if the user had shopped in person. Moreover, people who shop online for groceries also shop in‐person, and in‐person shopping levels are expected to return to prepandemic levels. Thus, online grocery shopping is not seen as a solution to reducing overall grocery shopping travel. It is nonetheless beneficial for retailers to keep online grocery shopping available and convenient, as we do find a significant portion of the population who reported wanting to continue grocery shopping online.

Policymakers may wish to consider how to increase access to online shopping. Specifically, more vulnerable populations, older people, and those without cars stand to benefit from the ability to have groceries delivered, at least some of the time. While online grocery shopping may cost more, for example due to delivery fees and tips for the delivery person, a major impediment is access to the Internet. Older and disabled individuals may not be able to use the online platforms needed to shop online. Carless households may benefit from access to healthier food options that are not available in their local neighborhood (i.e., they live in a food desert). So there are potential benefits in finding ways to provide means for more people to utilize online grocery shopping, such as by providing subsidies to carless households or disabled individuals, but ability to use internet platforms may be a more difficult problem to solve.

Future research in this area will need to focus in more detail on how grocery shopping behavior changed. Did people make fewer but larger purchases to minimize the frequency of trips? Did they increase frequency but make shorter trips with smaller purchases? What type of products were typically bought online versus in‐person? These issues are important for understanding the impacts on travel behavior, and issues such as the parking requirements at grocery stores or the impact on the proliferation of delivery vehicles in neighborhoods. We were unable to address these questions.

We acknowledge several limitations to our data and analysis. First, we only asked about online grocery shopping for delivery. ‘Curb side’ pick‐up became novel during the pandemic to encourage as little person‐to‐person interaction as possible without having to pay for a delivery fee. In that sense, there is no ‘before’ to analyze, since it did not exist. However, it is possible that people who responded to the survey answered that they had never ordered groceries for delivery, when in fact they had ordered it for pick‐up (albeit not at the curb). Second, we used an online panel aimed to be to be representative of New Jersey with respect to age, income, and gender, but not towards education. Hence, the conclusions that stemmed from our survey with respect to educational attainment cannot be applied to the entire state of New Jersey, and we also suffer from the normal caveats of an online panel, which is essentially a paid group of survey takers arranged by Qualtrics. Related to this, since the survey was administered online only, our sample would be more versed with the Internet, and possibly, with online shopping.

## Supporting information


**Table S1:** Binomial Regression Results for In Person Grocery Shopping Before, During and After the Pandemic
**Table S2:** Binomial Regression Results for Online Grocery Shopping Before, During, and After the Pandemic
**Table S3:** Response Rates for questions related to attitudes towards COVID‐19

## Footnotes

^1^

https://www.qualtrics.com/research-services/online-sample/ provides information on Qualtrics.

^2^
We also estimated similar models for grocery shopping behavior both before the pandemic and respondent expectations of grocery shopping behavior after the pandemic. These models are included as supplemental material.
