# Supplementary material for: Browsing for food: Will COVID‐induced online grocery delivery persist?
Source: Reg Sci Policy Prac. 2022 Jun 1:10.1111/rsp3.12542. Online ahead of print. doi: 10.1111/rsp3.12542 (PMC9347773; doi:10.1111/rsp3.12542)
Supplement: Supplementary file 1 — Table S1: Binomial Regression Results for In Person Grocery Shopping Before, During and After the Pandemic Table S2: Binomial Regression Results for Online Grocery Shopping Before, During, and After the Pandemic Table S3: Response Rates for questions related to attitudes towards COVID‐19 [file RSP3-9999-0-s001.docx]

**Supplementary Material for “*Browsing for food: Will COVID-induced on-line grocery delivery persist?”***

**Table S1** displays the regression results for frequent In-Person Grocery Shopping before, during and (expected) after the Pandemic. **Table S2** displays the regression results for frequent Online Grocery Shopping before, during, and (expected) after the pandemic. The tables supplement **Table 6** in the main document, which displays results for during the pandemic.

**Table S1: Binomial Regression Results for In Person Grocery Shopping Before, During and After the Pandemic**

|  | | | | |
| --- | --- | --- | --- | --- |
|  | Dependent variable: Shopped for Groceries in Person at least once a week | | |  |
|  |  |  |  |  |
|  | Before | During | After (expected) |  |
|  | (1) | (2) | (3) |  |
|  | | | |  |
| Female (Ref: Male) | -0.047 | **-0.219^*^** | **-0.235^**^** |  |
|  | (0.120) | **(0.130)** | **(0.119)** |  |
| Children Present | **0.318^**^** | -0.085 | 0.153 |  |
|  | **(0.131)** | (0.143) | (0.130) |  |
| **Education (Ref: High School or less)** |  |  |  |  |
| Associates | **0.439^**^** | 0.184 | 0.311 |  |
|  | **(0.206)** | (0.235) | (0.207) |  |
| Bachelors | 0.251 | **0.433^**^** | 0.230 |  |
|  | (0.155) | **(0.174)** | (0.156) |  |
| Masters | **0.438^**^** | **0.579^***^** | 0.141 |  |
|  | **(0.182)** | **(0.200)** | (0.183) |  |
| Doctoral Degree | **0.787^**^** | 0.306 | -0.044 |  |
|  | **(0.395)** | (0.411) | (0.386) |  |
| Professional Degree | 0.441 | 0.426 | -0.431 |  |
|  | (0.378) | (0.402) | (0.393) |  |
| **Income (Reference: <$35,000)** |  |  |  |  |
| 35,000- 49,999 | -0.147 | -0.141 | -0.099 |  |
|  | (0.244) | (0.277) | (0.245) |  |
| 50,000-74,999 | 0.142 | -0.091 | -0.020 |  |
|  | (0.190) | (0.217) | (0.191) |  |
| 75,000-99,999 | 0.318 | -0.009 | 0.178 |  |
|  | (0.202) | (0.227) | (0.203) |  |
| 75,000-99,999 | 0.296 | 0.092 | 0.263 |  |
|  | (0.230) | (0.252) | (0.230) |  |
| 125,000-149,999 | **0.651^***^** | 0.395 | **0.568^**^** |  |
|  | **(0.249)** | (0.263) | **(0.247)** |  |
| >150,000 | 0.319 | 0.063 | 0.296 |  |
|  | (0.213) | (0.235) | (0.213) |  |
| **Age Group (Reference: 18-24)** |  |  |  |  |
| 25-34 | 0.087 | 0.0004 | -0.057 |  |
|  | (0.212) | (0.231) | (0.209) |  |
| 35-49 | 0.157 | -0.017 | -0.235 |  |
|  | (0.214) | (0.236) | (0.213) |  |
| 50-64 | **0.656^***^** | 0.321 | 0.222 |  |
|  | **(0.218)** | (0.241) | (0.216) |  |
|  |  |  |  |  |
| > 65 | **0.798^***^** | 0.228 | 0.323 |  |
|  | **(0.281)** | (0.320) | (0.281) |  |
| **Race/Ethnicity** |  |  |  |  |
| Black | -0.240 | 0.146 | **-0.362^**^** |  |
|  | (0.157) | (0.173) | **(0.159)** |  |
| Asian | -0.235 | -0.057 | -0.078 |  |
|  | (0.206) | (0.230) | (0.205) |  |
| Hispanic | -0.135 | 0.178 | -0.084 |  |
|  | (0.154) | (0.166) | (0.154) |  |
| **Vehicles Per Person** | -0.085 | 0.029 | -0.045 |  |
|  | (0.127) | (0.135) | (0.127) |  |
| **Employment Type (Ref: Can work from home)** |  |  |  |  |
| Cannot Work from home | 0.107 | -0.066 | -0.172 |  |
|  | (0.141) | (0.151) | (0.140) |  |
| Not Employed | -0.058 | **-0.376^*^** | **-0.453^**^** |  |
|  | (0.178) | **(0.196)** | **(0.178)** |  |
| **Attitudes towards COVID-19 Pandemic** |  |  |  |  |
| Believers | **0.261^**^** | 0.136 | **0.216^**^** |  |
|  | **(0.109)** | (0.117) | **(0.109)** |  |
| Deniers | **0.170^**^** | **0.534^***^** | **0.181^***^** |  |
|  | **(0.068)** | **(0.073)** | **(0.068)** |  |
| Fearful | -0.059 | -0.065 | -0.059 |  |
|  | (0.103) | (0.112) | (0.103) |  |
| Constant | -0.840^***^ | -1.096^***^ | -0.139 |  |
|  | (0.275) | (0.299) | (0.271) |  |
|  | | | |  |
| Observations | 1,395 | 1,395 | 1,394 |  |
| Log Likelihood | -914.932 | -797.714 | -916.374 |  |
| Akaike Inf. Crit. | 1,883.865 | 1,649.428 | 1,886.748 |  |
| TJUR R2 | 0.070 | 0.093 | 0.055 |  |
| Note: | ^*^p<0.10; ^**^p<0.05; ^***^p<0.01 | | |  |

| Table S2: Binomial Regression Results for Online Grocery Shopping Before, During, and After the Pandemic | | | |
| --- | --- | --- | --- |
|  | Dependent variable: Shopped Online for Groceries at least Once a Month | | |
|  |  | | |
|  | Before | During | After (expected) |
|  | (1) | (2) | (3) |
|  | | | |
| Female (Ref: Male) | **-0.457^***^** | 0.060 | -0.230 |
|  | **(0.144)** | (0.148) | (0.150) |
| Children present | **0.695^***^** | **0.467^***^** | 0.240 |
|  | **(0.151)** | **(0.156)** | (0.159) |
| **Education (Ref: High School or less)** |  |  |  |
| Associates | -0.165 | **0.440^*^** | 0.349 |
|  | (0.272) | **(0.256)** | (0.263) |
| Bachelors | 0.144 | **0.397^**^** | 0.248 |
|  | (0.199) | **(0.192)** | (0.196) |
| Masters | **0.433^*^** | 0.311 | **0.442^*^** |
|  | **(0.224)** | (0.225) | **(0.228)** |
| Doctoral degree | 0.415 | **1.260^**^** | **1.176^**^** |
|  | (0.442) | **(0.537)** | **(0.533)** |
| Professional Degree | 0.493 | 0.652 | 0.301 |
|  | (0.442) | (0.451) | (0.479) |
| **Income (Ref: <$35,000)** |  |  |  |
| 35,000- 49,999 | -0.174 | 0.290 | 0.268 |
|  | (0.305) | (0.302) | (0.309) |
| 50,000-74,999 | **-0.436^*^** | 0.137 | 0.102 |
|  | **(0.244)** | (0.233) | (0.239) |
| 75,000-99,999 | -0.057 | 0.264 | 0.304 |
|  | (0.250) | (0.246) | (0.253) |
| 100,000-124,999 | 0.021 | 0.264 | 0.158 |
|  | (0.274) | (0.280) | (0.286) |
| 125,000-149,999 | 0.126 | **0.914^***^** | **0.536^*^** |
|  | (0.292) | **(0.314)** | **(0.315)** |
| >150,000 | -0.153 | 0.386 | **0.590^**^** |
|  | (0.258) | (0.261) | **(0.266)** |
| **Age Group (Ref: 18-24)** |  |  |  |
| 25-34 | 0.353 | 0.110 | 0.232 |
|  | (0.228) | (0.240) | (0.245) |
| 35-49 | -0.028 | -0.109 | -0.178 |
|  | (0.236) | (0.248) | (0.252) |
| 50-64 | **-0.966^***^** | -0.376 | **-0.655^**^** |
|  | **(0.267)** | (0.258) | **(0.265)** |
| >65 | **-1.427^***^** | -0.386 | -0.382 |
|  | **(0.435)** | (0.364) | (0.370) |
| **Race/Ethnicity** |  |  |  |
| Black | 0.144 | 0.299 | **0.488^**^** |
|  | (0.185) | (0.189) | **(0.195)** |
| Asian | **-0.663^**^** | 0.145 | -0.141 |
|  | **(0.264)** | (0.252) | (0.258) |
| Hispanic | 0.179 | 0.232 | **0.509^***^** |
|  | (0.174) | (0.183) | **(0.186)** |
| Vehicles Per Person | **-0.301^**^** | **-0.420^***^^[[1]](#footnote-1)^** | -0.231 |
|  | **(0.149)** | **(0.155)** | (0.155) |
| **Employment Type (Ref: Can work from home)** |  |  |  |
| Cannot Work from home | **-1.111^***^** | **-0.599^***^** | **-0.530^***^** |
|  | **(0.161)** | **(0.170)** | **(0.172)** |
| Not Employed | -0.328 | **-0.451^**^** | -0.204 |
|  | (0.204) | **(0.211)** | (0.216) |
| **Attitudes towards COVID-19 Pandemic** |  |  |  |
| Believers | 0.182 | **0.283^**^** | 0.214 |
|  | (0.130) | **(0.135)** | (0.136) |
| Deniers | **0.491^***^** | **0.203^**^** | **0.358^***^** |
|  | **(0.079)** | **(0.086)** | **(0.087)** |
| Fearful | 0.077 | **0.216^*^** | 0.143 |
|  | (0.125) | **(0.129)** | (0.130) |
| **Previous Shopping Experience** |  |  |  |
| Never Shopped Online Before the Pandemic |  | **-2.403^***^** | **-2.383^***^** |
|  |  | **(0.152)** | **(0.153)** |
| Constant | -0.099 | 0.718^**^ | 0.663^**^ |
|  | (0.311) | (0.325) | (0.330) |
| Observations | 1,392 | 1,391 | 1,390 |
| Log Likelihood | -665.763 | -653.832 | -634.190 |
| Akaike Inf. Crit. | 1,385.525 | 1,363.663 | 1,324.381 |
| TJUR R2 | 0.26 | 0.38 | 0.40 |
| Note: | ^*^p<0.10; ^**^p<0.05; ^***^p<0.01 | | |

**Table S3** below provides the response rates for **Table 4** in the main manuscript. The questions are used in the Factor Analysis described in Section 3.4 of the manuscript.

Table S3: Response Rates for questions related to attitudes towards COVID-19

| **Variable** | **Sample n** | **Sample %** | N |
| --- | --- | --- | --- |
| **I am concerned that if I catch the coronavirus, I will be very ill:** |  |  | 1417 |
| Totally disagree | 118 | 8.33% |  |
| Somewhat disagree | 183 | 12.90% |  |
| Neither agree nor disagree | 238 | 16.80% |  |
| Somewhat agree | 424 | 29.90% |  |
| Totally agree | 454 | 32.00% |  |
| **I am concerned that friends or family members will be very ill if they catch the coronavirus:** |  |  | 1417 |
| Totally disagree | 75 | 5.29% |  |
| Somewhat disagree | 108 | 7.62% |  |
| Neither agree nor disagree | 236 | 16.70% |  |
| Somewhat agree | 459 | 32.40% |  |
| Totally agree | 539 | 38.00% |  |
| **Everyone should just stay home as much as possible until the coronavirus has subsided:** |  |  | 1418 |
| Totally disagree | 84 | 5.92% |  |
| Somewhat disagree | 130 | 9.17% |  |
| Neither agree nor disagree | 253 | 17.80% |  |
| Somewhat agree | 387 | 27.30% |  |
| Totally agree | 564 | 39.80% |  |
| **Society is overreacting to the coronavirus:** |  |  | 1417 |
| Totally disagree | 528 | 37.30% |  |
| Somewhat disagree | 221 | 15.60% |  |
| Neither agree nor disagree | 261 | 18.40% |  |
| Somewhat agree | 235 | 16.60% |  |
| Totally agree | 172 | 12.10% |  |
| **Shutting down businesses to prevent the spread of coronavirus is not worth the economic damage that will result:** |  |  | 1417 |
| Totally disagree | 209 | 14.70% |  |
| Somewhat disagree | 286 | 20.20% |  |
| Neither agree nor disagree | 341 | 24.10% |  |
| Somewhat agree | 326 | 23.00% |  |
| Totally agree | 255 | 18.00% |  |
| **My friends and family expect me to stay at home until the coronavirus subsides:** |  |  | 1418 |
| Totally disagree | 115 | 8.11% |  |
| Somewhat disagree | 186 | 13.10% |  |
| Neither agree nor disagree | 388 | 27.40% |  |
| Somewhat agree | 431 | 30.40% |  |
| Totally agree | 298 | 21.00% |  |
| **Everyone should wear a face mask when they are near other people:** |  |  | 1417 |
| Totally disagree | 44 | 3.11% |  |
| Somewhat disagree | 72 | 5.08% |  |
| Neither agree nor disagree | 179 | 12.60% |  |
| Somewhat agree | 231 | 16.30% |  |
| Totally agree | 891 | 62.90% |  |

1. even if we exclude the “never shopped online before” variable and have all the variables be the same for comparison purposes, the coefficient for vehicles per person is still higher (-0.334). [↑](#footnote-ref-1)
